# Members of the tomato *FRUITFULL* MADS-box family regulate style abscission and fruit ripening

**DOI:** 10.1093/jxb/eru137

**Published:** 2014-04-10

**Authors:** Shufen Wang, Gang Lu, Zheng Hou, Zhidan Luo, Taotao Wang, Hanxia Li, Junhong Zhang, Zhibiao Ye

**Affiliations:** Key Laboratory of Horticultural Plant Biology, Ministry of Education, Huazhong Agricultural University, Wuhan 430070, China

**Keywords:** Development, fruit shape, FRUITFULL, MADS-box, ripening, tomato.

## Abstract

The tomato (*Solanum lycopersicum*) protein MADS-RIN plays important roles in fruit ripening. In this study, the functions of two homologous tomato proteins, FUL1 and FUL2, which contain conserved MIKC domains that typify plant MADS-box proteins, and which interact with MADS-RIN, were analysed. Transgenic functional analysis showed that *FUL1* and *FUL2* function redundantly in fruit ripening regulation, but exhibit distinct roles in the regulation of cellular differentiation and expansion. Over-expression of *FUL2* in tomato resulted in a pointed tip at the blossom end of the fruit, together with a thinner pericarp, reduced stem diameter, and smaller leaves, but no obvious phenotypes resulted from *FUL1* over-expression. Dual suppression of *FUL1* and *FUL2* substantially inhibited fruit ripening by blocking ethylene biosynthesis and decreasing carotenoid accumulation. In addition, the levels of transcript corresponding to *ACC SYNTHASE2* (*ACS2*), which plays a key role in ethylene biosynthesis, were significantly decreased in the *FUL1/FUL2* knock-down tomato fruits. Overall, our results suggest that FUL proteins can regulate tomato fruit ripening through fine-tuning ethylene biosynthesis and the expression of ripening-related genes.

## Summary

The *FRUITFULL* genes regulate tomato fruit ripening in a redundant manner. They are functional diversely in fruit shape regulation through affecting cellular differentiation and expansion.

## Introduction

Members of MADS-box transcription factor families, which are widely distributed among animal and plant taxa, contain a conserved domain of approximately 60 amino acids in the N-terminal region, named the MADS-box (M) domain. Plant MADS-box gene families have been studied in detail and are now associated with a wealth of genetic and evolutionary data (Ferrario *et al.*, 2004). In addition to the M domain, most of the plant MADS-box proteins contain three other domains: the Intervening (I) domain, the Keratin (K) domain, and the C-terminal (C) domain (Kaufmann *et al.*, 2005). The MADS-box domain is involved in DNA binding and dimerization, while the I domain is responsible for DNA-binding specificity during dimer formation and the K domain mediates protein–protein interactions. Compared with the other domains, the C domain shows less sequence conservation and it has been found to be essential for ternary complex formation and transcriptional activation (Davies *et al.*, 1996; Riechmann *et al.*, 1996; Riechmann and Meyerowitz, 1997; Kramer *et al.*, 1998; Cho *et al.*, 1999; Messenguy and Dubois, 2003; de Folter and Angenent, 2006).

Plant MADS-box genes were first investigated in the context of functional studies of *Arabidopsis* floral organ development and flowering time (Sommer *et al.*, 1990; Yanofsky *et al.*, 1990) and are the major modules in the well-known ABCDE model of floral organs. In *Arabidopsis*, the counterparts of MADS-box genes are: class A, *APETALA1* (*AP1*); class B, *PISTILATA* (*PI*) and *AP3*; class C, *AGAMOUS* (*AG*); class D, *SEEDSTICK*/*AGAMOUS-LIKE11* (*STK*/*AGL11*); and class E, *SEPALLATA* (*SEP1*, *SEP2*, *SEP3*, and *SEP4*). Moreover, some other MADS-box genes, including *SOC1*, *FLC*, *AGL24*, and *SVP*, have been confirmed to be involved in flowering time and flower initiation (Michaels and Amasino, 1999; Hartmann *et al.*, 2000; Hepworth *et al.*, 2002; Yu *et al.*, 2002; Michaels *et al.*, 2003; Searle *et al.*, 2006; Kim *et al.*, 2007; Liu *et al.*, 2008). MADS-box genes also function in seed pigmentation and endothelium development (*TRANSPARENT TESTA16*) (Nesi *et al.*, 2002), root development (*AGL12* and *AGL17*) (Rounsley *et al.*, 1995; Tapia-Lopez *et al.*, 2008), and fruit formation (*SHP1*, *SHP2*, and *FUL*) (Ferrandiz *et al.*, 2000; Gu *et al.*, 1998; Liljegren *et al.*, 2000).

Other than these *Arabidopsis* genes, another well studied MADS-box gene is *MADS-RIN*, which was identified after screening a series of tomato mutants (Vrebalov *et al.*, 2002). Fruits from the loss-of-function *rin* (*ripening inhibitor*) mutant exhibit perturbed ripening, as shown by the absence of a respiratory climacteric and associated ethylene evolution, as well as reduced softening, carotenoid accumulation, and the production of flavour compounds. While *rin* fruits are sensitive to ethylene, their ripening is not induced by exogenous ethylene (Vrebalov *et al.*, 2002). In strawberry, a non-climacteric fruit, silencing of a tomato *MADS-RIN* homologue, *FaMADS9*, also leads to the inhibition of normal development and ripening (Seymour *et al.*, 2011). These results suggest that *MADS-RIN* genes play a conserved function in ripening regulation in both climacteric and non-climacteric fruits (Seymour *et al.*, 2011).

The mechanism by which MADS-RIN proteins regulate fruit ripening has been studied and it was reported that the tomato MADS-RIN protein influences gene expression by binding to CArG box sequences in the promoter regions of many ripening-related genes (Martel *et al.*, 2011; Fujisawa *et al.*, 2012; Qin *et al.*, 2012). MADS-domain proteins can form hetero/homo-dimers, or higher order complexes, through protein–protein interactions (Favaro *et al.*, 2002; Shchennikova *et al.*, 2004; de Folter and Angenent, 2006) and these associations are essential for their function (Leseberg *et al.*, 2008). A yeast two-hybrid screen of a prey library constructed from red ripe tomato fruit, with MADS-RIN as the bait, identified several other MADS-box proteins, including TDR4/FUL1, MBP7/FUL2, TAGL1, TAG1, MBP21, and TDR5 as putative MADS-RIN protein interactors (Martel *et al.*, 2011). Similar to MADS-RIN, one of these proteins, TAGL1, also functions in tomato fruit ripening regulation and *TAGL1*-RNAi transgenic lines showed defects in fruit ripening (Vrebalov *et al.*, 2009). This result suggests that MADS-RIN forms complexes with other proteins to control fruit ripening.

The interaction partners of MADS-RIN, TDR4/FUL1 and MBP7/FUL2, are close homologues of the *Arabidopsis* protein FRUITFULL, which has been suggested to regulate the transcription of genes required for cellular differentiation during fruit development (Gu *et al.*, 1998). In addition, *VmTDR4*, a *FRUITFULL* homologue from bilberry (*Vaccinium myrtillus*) was found to regulate anthocyanin biosynthesis during fruit ripening (Jaakola *et al.*, 2010). These results suggest that *FUL1* and *FUL2* might similarly play important roles during tomato fruit development, a hypothesis that was tested in this current study through the functional analysis of transgenic plants. As reported by Bemer and by Burko, it was confirmed that *FUL1* and *FUL2* played redundant roles in tomato fruit pigmentation accumulation and that *FUL2* was involved in tomato leaf development (Bemer *et al.*, 2012; Burko *et al.*, 2013). Furthermore, our data showed that *FUL1* and *FUL2* play distinct roles in regulating cellular differentiation and expansion. The over-expression of *FUL2*, but not *FUL1*, affected fruit shape, resulted in a thinner fruit pericarp, and changed the number of layers of cells in the stem. These results demonstrate that *FUL* MADS-box transcription factors have diverse functions in growth and developmental regulation in tomato.

## Materials and methods

### Plant material and growth conditions

All tomato (*Solanum lycopersicum* cv. Ailsa Craig) plants, including transgenic lines and a mutant line homozygous for the *rin* mutation in the Ailsa Craig background, were grown in a glasshouse under natural daylight and with 60–75% relative humidity and ambient temperature (>20 °C). The tomato transgenic lines were advanced to the T_2_ generation. Flowers were tagged at the full-bloom stage to synchronize developmental comparisons. For analysis, 1, 7, and 14 days post-anthesis (DPA) corresponded to ovaries of 1, 7, and 14 DPA, respectively. The fruit stages used were immature green (IG), mature green (MG), breaker (BR), yellow ripe (YR), and red ripe (RR), which were picked at approximate 28, 35, 38, 41, and 44 DPA, respectively.

### Construct recombination and plant transformations

For the *FUL1*/*FUL2* double-silencing construct, a 362bp fragment of the *FUL1*-IK domain was amplified by *FUL1* sequence-specific primers (amplified with the primers in Supplementary Table S1 at *JXB* online). Fragments were incorporated into the pDONR221 vectors using the Clonase BP reaction (Invitrogen). The LR reaction (Invitrogen) was performed subsequently to incorporate the fragments into the pHELLSGATE8 vector. For the *FUL1* and *FUL2* over-expression constructs, the coding regions of *FUL1* and *FUL2* were amplified and cloned into the *Xba*I and *Kpn*I sites of the pMV2 vector (modified from pHELLSGATE2). The expressions of transgene were driven using the CaMV 35S promoter in both vector systems.

For the Pro_*FUL2*_::GUS construct, a genomic DNA (gDNA) sequence (from –2518 to –24bp) upstream of the *FUL2* coding sequence was amplified using sequence-specific primers. The primers were fused with the attB1 and attB2 sites for recombination. The resulting fragment was recombined into the pDONR221 vector using BP recombinase and the LR reaction was performed to incorporate the fragment into the pV3P vector (modified from pHELLSGATE2) which contains the glucuronidase synthase (GUS) coding sequences. All of the recombinant constructs were transformed into the *Agrobacterium* strain C58 by electroporation, and subsequently transformed into tomato cotyledon explants.

### Ethylene assay

To measure ethylene production, BR stage fruits of approximately the same size were harvested and kept in sealed containers for 4h and left open for 20h each day (16 d) at room temperature (each biological replicate contained three fruits). Ethylene was measured in the headspace of the sealed containers by sampling with a syringe. Measurements were performed as described by Vrebalov *et al.* (2009). All samples involved three technical and three biological replicates.

### Gene expression analysis

RNA from various tissues of wild-type and transgenic plants were isolated using TRIzol^®^ 117 reagent (Invitrogen, USA). For cDNA synthesis, 3 µg RNA was used with M-MLV reverse transcriptase (Toyobo, Japan) according to the manufacturer’s instructions. The cDNA concentrations were normalized to actin expression levels for RT-PCR analysis. Primers of the ripening-related genes are listed in Supplementary Table S1 at *JXB* online. The actin gene was used as an internal control for quantitative real-time PCR (qPCR), which was performed using the Power SYBR Premix Ex Taq kit and the TaKaRa two-step method (TaKaRa, Japan). PCR products were quantified using the Roche LightCycler 480 Real-Time PCR Detection System and the SYBR Green I Master Kit (Roche, Switzerland). The wild-type and transgenic plants were represented by three biological replicates for each sample.

### Paraffin section

Light microscopic observation of paraffin sections was used to measure the number of cell layers and cell sizes in stems and pericarp of both wild-type and transgenic plants. For the fruit material, 14 DPA and BR stage fruits were harvested and at least nine pericarp sections were isolated. For the stem samples, the 3rd, 13th, and 17th internodes were selected and at least nine sections were measured and harvested for analysis. Paraffin sections were prepared as described by Yang *et al.* (2011) and the number of cell layers was counted manually. Photomicrographs were taken using an Olympus microscope.

### GUS staining

Slices of fruits and stems from the transgenic lines transformed with the native promoter of *FUL2*-driving expression of the GUS gene (Pro_*FUL2*_::GUS) were stained with a GUS staining solution (100mM sodium phosphate buffer) to evaluate GUS activity. Staining was allowed to proceed for 5h at 37 °C in the dark and then washed with a graded ethanol series at room temperature for decolorization and observation by light microscopy (OLYMPUS SZX12).

## Results

### *FUL1* and *FUL2* show distinct expression patterns in tomato tissues

**Fig. 1.**
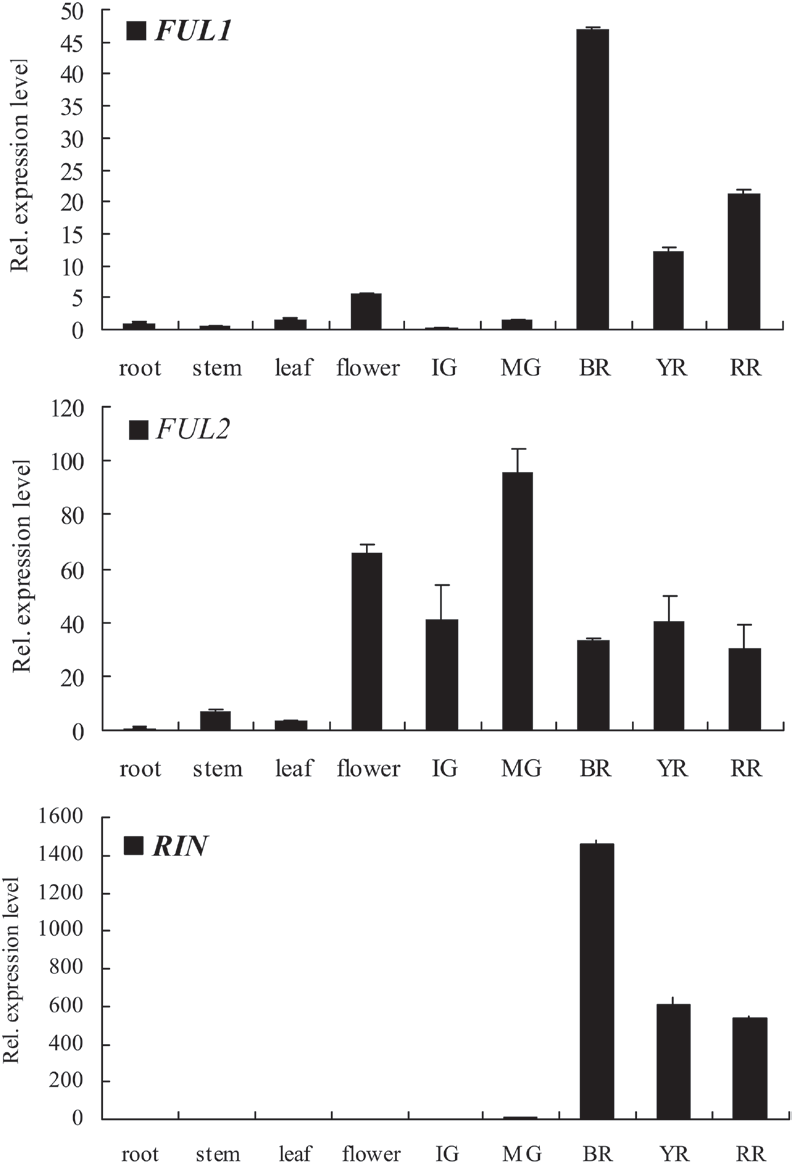
Expression patterns of *FUL1, FUL2*, and *RIN* in wild-type (Ailsa Craig) tissues of tomato. The relative expression levels of each gene in different tissues were compared against its root level. IG, Immature Green; MG, Green Ripe; BR, Breaker; YR, Yellow Ripe; RR: Red Ripe (*n*=3). Standard errors are shown.

Previous studies indicate that expression of *MADS-RIN* is induced coincident with ripening and peaks at the red ripe stage (Vrebalov *et al.*, 2002). For comparative purposes, real-time quantitative PCR (qPCR) was performed to investigate the expression levels of *FUL1*, *FUL2*, and *RIN* in different tissues and fruit developmental stages of the tomato cultivar Ailsa Craig (Fig. 1). The results showed that the expression of *FUL1* was similar to *RIN*, and was very low in roots, stems, leaves, and in the early stages of fruit development, but increased rapidly at the breaker (BR) stage, which marked the onset of ripening. This suggested that *FUL1* might have a similar function to *RIN* and regulate fruit ripening. On the other hand, the expression of *FUL2* was high in flowers and throughout fruit development, as well as in stems and leaves. Indeed, *FUL2* was expressed in all tissues tested except roots, indicating that it functions in both reproductive and vegetative organs. It was noted that Bemer and colleagues reported similar expression patterns for *FUL1* and *FUL2* (Bemer *et al.*, 2012). Taken together, the contrasting spatial and temporal expression patterns of *FUL1* and *FUL2* indicated that *FUL1* and *FUL2* may have distinct functions in the regulation of tomato plant growth.

### Over-expression of tomato *FUL2* inhibits the abscission of styles from fruit leading to a ‘pointed tip’ phenotype

**Fig. 2.**
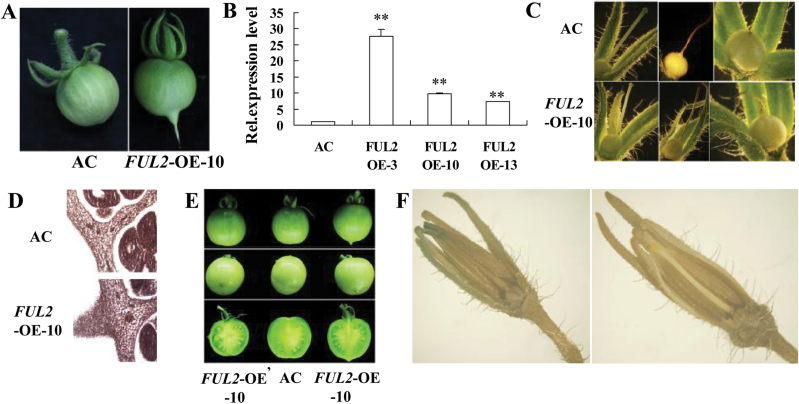
Over-expression of *FUL2* leading to a pointed tip phenotype. (A) *FUL2*-OE-10 fruit exhibited a pointed tip phenotype, in contrast to wild-type fruit. (B) The relative expression levels of *FUL2* in the *FUL2*-OE lines compared with the wild-type (*n=3*). (C) Ultrastructure of ovaries from *FUL2*-OE-10 and the wild type. Left panel, ovary 1 d post-anthesis; middle panel, ovary 7 d post-anthesis; right panel, ovary 14 d post-anthesis. (D) Stereomicroscope comparison of ovaries from *FUL2*-OE-10 and wild-type plants at 2 weeks after anthesis. (E) Phenotype of *FUL2*-OE-10 transgenic fruit. *FUL2*-OE’-10 and *FUL2*-OE-10, over-expression transgenic fruit with or without the removal of the stigma at the flowering stage. (F) Histochemical localization of GUS activity in the flowers of transgenic tomato. Pro_*FUL2*_::GUS (left) and wild-type (right) plants. (This figure is available in colour at *JXB* online.).

To gain an insight into the functions of *FUL1* and *FUL2*, vectors were constructed to mediate their suppression (RNAi) or over-expression and transformed into tomato (cv. Ailsa Craig). At least 15 independent transgenic lines were obtained corresponding to each vector. Nine of the 15 *FUL2* over-expressing transgenic lines showed an obvious alteration in fruit shape with a prominent pointed tip at the blossom end of the fruit (Fig. 2A), of which three lines (FUL2-OE-3, 10, and 13) were selected for transcriptional analysis. Expression of *FUL2* in fruit of these lines was 7–28-fold higher than that in the wild type (Fig. 2B). The developmental onset of the pointed tip phenotype was assessed using stereomicroscopy and at 1 DPA, an early stage of fruit development, styles and ovaries of the *FUL2*-OE and the wild type showed no visible differences. Subsequently, at 7 DPA, the style of the wild type typically wilted from the tip to the base and abscised while the *FUL2*-OE lines showed a similar abscission phenotype, but with a visibly enlarged base. However, by 14 DPA, rather than showing abscission, as is typical of the wild type, the style of *FUL2*-OE was still connected to the ovary and the visibly enlarged basal region developed into a pointed tip (Fig. 2C), suggesting that this phenotype of *FUL2*-OE fruit is related to an abnormally persistent style.

To evaluate the abnormal style development further, a series of paraffin sections of ovaries at different developmental stages was prepared for microscopic observation. While the intact style typically abscised from the wild-type fruit, the style of *FUL2*-OE remained attached to the swollen ovary (young fruit) at 14 DPA with no obvious abscission zone (AZ) formation, and normal cell development was observed in the pointed tip (Fig. 2D). These results suggested that *FUL2* may regulate style abscission by influencing ovary cell development, rather than AZ formation. In order to confirm that the abnormal development was the principal cause of the pointed tip phenotype, the growth was compared of *FUL2*-OE fruits from which the style had been removed following pollination and fertilization or had been left in place. Two weeks after pollination, no pointed tip was observed on the style-removed *FUL2*-OE fruits while, as expected, the non-treated *FUL2*-OE had the pointed tip phenotype (Fig. 2E). These results indicated that *FUL2* affected style abscission by regulating ovary development, resulting in a pointed tip phenotype.

To obtain further support for this conclusion, the expression of *FUL2* during fruit set was investigated using the GUS reporter gene driven by the presumed *FUL2* promoter in transgenic tomato plants (Pro_*FUL2*_::GUS). GUS staining of 1 DPA Pro_*FUL2*_::GUS flowers showed that the *FUL2* promoter had high expression activity in sepals and the stigma. Within the style, GUS activity was detected in the upper portion but not the base (Fig. 2F). This suggested that *FUL2* negatively regulates the abscission of the pistil from tomato fruit at an early developmental stage and that reducing its expression in the pistil after pollination and fertilization is necessary to promote pistil abscission.

### Over-expression of *FUL2* suppresses tomato shoot growth

**Fig. 3.**
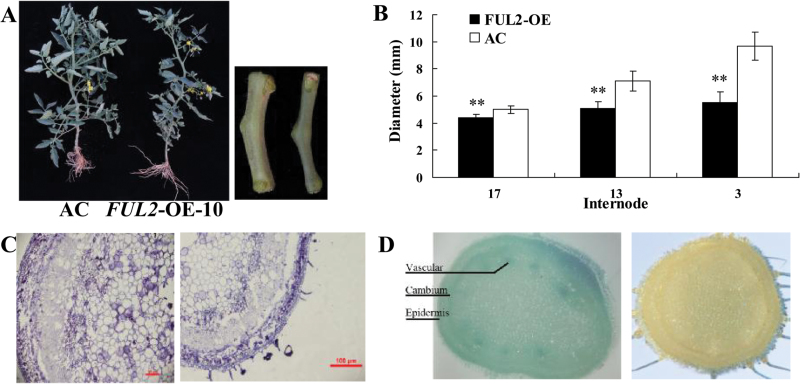
Over-expression of *FUL2* results in a thinner stem. (A) Phenotypes of *FUL2*-OE-10 (right) and the wild type (left). (B) Comparison of stem diameters of *FUL2*-OE and wild-type plants. (C) Transverse sections of stems from *FUL2*-OE-10 (right) and wild-type (left) plants. Scale bars=100mm. (D) Histochemical localization of GUS activity in the stem of transgenic tomato. Pro_*FUL2*_::GUS (left) and AC (right) plants. (This figure is available in colour at *JXB* online.).

In addition to the fruit pointed tip phenotype, it was observed that the shoots of the *FUL2*-OE lines had smaller leaves and thinner stems than those of wild-type plants (Fig. 3A). In order to understand better how *FUL2* influences main stem growth, the diameters of *FUL2*-OE main stems at different internodes were measured. Young stem diameters (the 17th internode) showed no obvious difference between *FUL2*-OE and wild-type plants, but stems of *FUL2*-OE plants were substantially narrower than those of the wild type in older regions. This difference increased with age and was approximately 5mm at the 3rd internode (Fig. 3B). Moreover, the average cell size in the stem at the 3rd internode of *FUL2*-OE plants was smaller (Fig. 3C), indicating that *FUL2* may block cell expansion in the stem during shoot growth.

In order to elucidate further the regulation of cell expansion in the stem by *FUL2*, GUS expression was assessed in transverse sections of the 17th stem internodes of Pro_*FUL2*_::GUS transgenic lines. GUS staining revealed that the *FUL2* gene showed no obvious expression in the pith, but high expression in the epidermis, cambium, and vascular tissues (Fig. 3D). The cambium is a layer of actively dividing cells and this cell division determines stem diameter. These results therefore support the idea that *FUL2* regulates stem growth by repressing cell division in the cambium and, consequently, the number of cell layers. Taken together, our data indicate that *FUL2* controls shoot growth by regulating both cell expansion and cell division.

### Over-expression of *FUL2* alters tomato pericarp structure

**Fig. 4.**
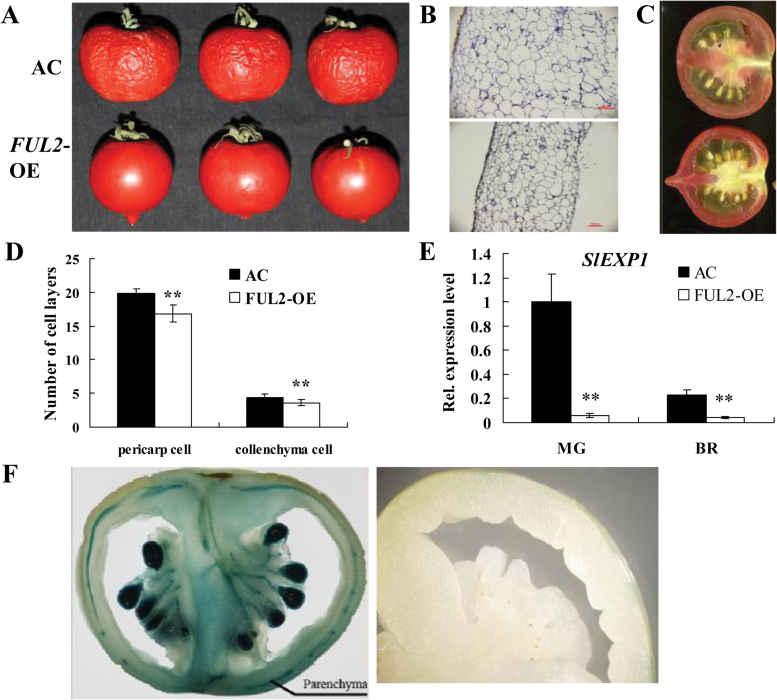
Altered pericarp structure in *FUL2* over-expressing fruit. (A) The phenotypes of *FUL2*-OE and wild-type fruits stored for 45 d. (B) Pericarp of *FUL2*-OE (bottom) and wild-type fruits (upper). (C) Comparison of the fruit pericarp longitudinal sections of *FUL2*-OE (bottom) and wild-type fruit (upper). (D) Comparison of pericarp cell layers between *FUL2*-OE and wild-type fruit. (E) Relative expression levels of *SlEXP1* in *FUL2*-OE and wild-type fruits. MG, Green Ripe; BR, Breaker (*n*=3). (F) Histochemical localization of GUS activity in the MG fruit of transgenic tomato. Pro_*FUL2*_::GUS (left) and AC fruit (right).

Shelf life is an important agronomic trait for many species of fleshy fruits, and one of the notable characteristics of the tomato *rin* mutant is substantially extending fruit shelf life (Giovannoni, 2007). To determine whether FUL2 potentially interacts with RIN to regulate tomato fruit shelf life, a storage experiment was performed with *FUL2*-OE fruits. Both wild-type and *FUL2*-OE fruits with peduncles were harvested at the BR stage and stored at room temperature for 45 d. As expected, *FUL2*-OE fruits dehydrated much more slowly than those of the wild type (Fig. 4A). A previous study suggested that silencing *FUL1*/*FUL2* expression in MicroTom fruit influences the expression of genes related to cuticle biosynthesis and post-harvest water loss, but does not affect pericarp development (Bemer *et al.*, 2012). Our results further suggest that *FUL2*, but not *FUL1*, functions in modulating post-harvest water loss.

In addition to the slower post-harvest dehydration, *FUL2*-OE fruits showed the thinner pericarp. The smaller size and the fewer number of pericarp cells were observed by using paraffin wax in the *FUL2*-OE pericarp (Fig. 4B, C, D). Moreover, this indicated that over-expressing *FUL2* inhibited pericarp cell expansion, possibly through a similar mechanism to that in the stem. In accordance with the high expression of *FUL2* in the stem, it was also highly expressed in parenchyma cells of the fruit pericarp (Fig. 4F). These results suggested that *FUL2* functions in cell division and expansion in both vegetative and reproductive organs. Plant-cell-wall-modifying proteins known as expansins have been reported to play roles in fruit softening and organ abscission, as well as affecting organ size and morphology by regulating cell growth (Brummell *et al.*, 1999; Cho and Cosgrove, 2000). Given the reduced cell size of the *FUL2*-OE fruit, it was investigated whether the expression of the *SlEXP1*, an expansin gene that is abundantly and specifically expressed during fruit ripening (Brummell *et al.*, 1999), was influenced by *FUL2*. It was determined that, indeed, the transcript levels of *SlEXP1* were significantly reduced in *FUL2*-OE fruit at the mature green (MG) and BR stages (Fig. 4E).

### Double silencing of *FUL1* and *FUL2* suppressed tomato fruit ripening

**Fig. 5.**
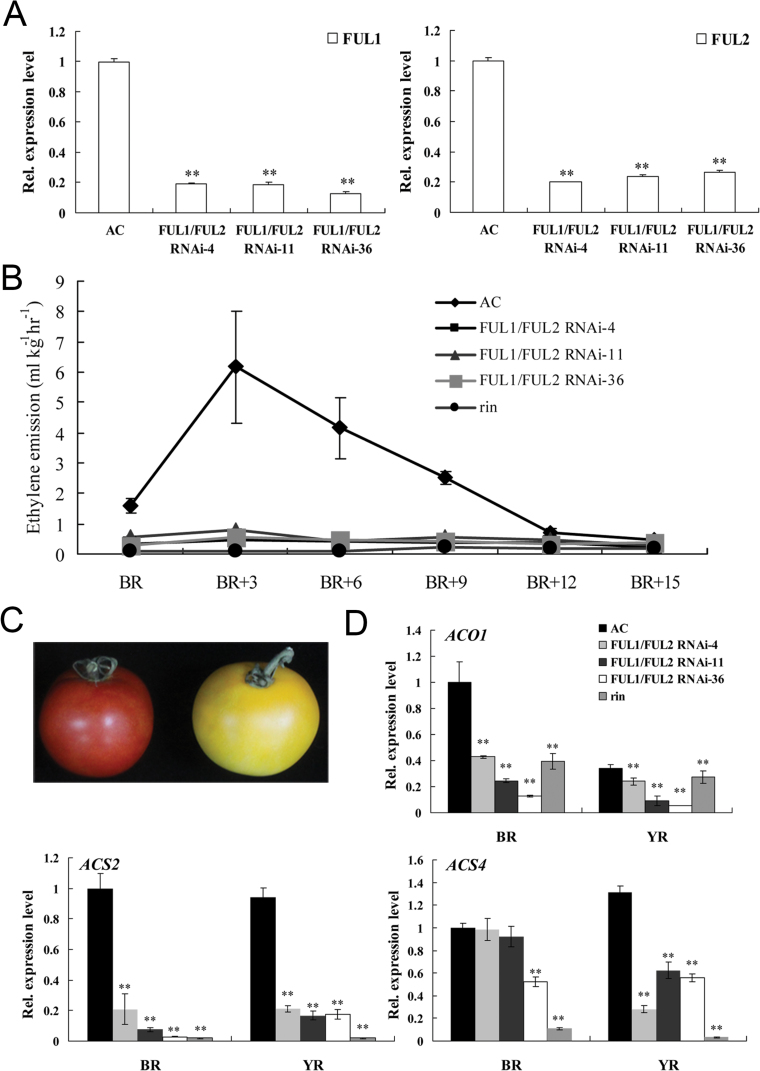
*FUL1*/*FUL2* regulated ethylene production. (A) The relative expression levels of *FUL1* and *FUL2* in the *FUL1*/*FUL2* RNAi fruits compared with the wild type (*n*=3). (B) Ethylene produced by of *FUL1*/*FUL2* RNAi and wild-type fruits at different development stages after the BR stage. (C) Wild-type (left) and *FUL1*/*FUL2* RNAi (right) fruits showed differences in fruit colour (BR+6 stage). (D) Quantitative real-time PCR analysis of ethylene biosynthesis related genes in *FUL1*/*FUL2* RNAi and wild-type fruits at the BR and YR stages.

Since *FUL1* and *FUL2* share a high degree of sequence similarity, and a high expression level in the pericarp during ripening, it was hypothesized that they might have similar functions. To test this, a sequence domain that is conserved between the FUL1 and FUL2 proteins was used as the basis to develop a strategy to doubly silence *FUL1*/*FUL2* by RNAi. Screening of the resulting transgenic lines indicated that this approach effectively suppressed the expression of both *FUL1* and *FUL2* (Fig. 5A).

As previously described by Bemer *et al.* (2012) the *FUL1*/*FUL2* doubly silenced transgenic lines showed bright yellow fruit during ripening (Fig. 5C). However, unlike the previous report, it was observed that fruits from the *FUL1*/*FUL2* doubly silenced lines showed blocked ethylene production compared with the wild type, which had a typical peak (6ml kg^–1^ h^–1^) at BR+3 d. The pattern of ethylene biosynthesis by the transgenic fruits was similar to that of the *rin* mutant, which produced <1ml kg^–1^ h^–1^ (Fig. 5B). The discrepancy between our results and those of Bemer *et al.* (2012) may be explained by differences in genotype, since Ailsa Craig was used here, while Bemer *et al.* (2012) used the dwarf mutant MicroTom as experimental material.

The climacteric burst of ethylene production in tomato fruit is largely driven by the ethylene biosynthetic genes *ACO1*, *ACS2*, and *ACS4* (Barry and Giovannoni, 2007), and their transcription is inhibited by the *rin* mutation (Martel *et al.*, 2011). The transcript levels of these genes were evaluated in the *FUL1*/*FUL2* doubly silenced fruit to establish whether this might explain the reduced ethylene production. Compared with wild-type fruit, the transcript abundance of *ACS2* in the transgenic fruit was reduced by 10-fold and 5-fold at the BR and YR stages, respectively, and *ACO1* showed a similar reduction (Fig. 5D). By contrast, transcript levels of *ACS4* showed no significant suppression at the BR stage, but a sharp decrease at the YR stage. Recently, Shima and colleagues also confirmed that both FUL1 and FUL2 can bind to the CArG-box sites of the *ACS2* promoter in a RIN-dependent manner (Shima *et al.*, 2013). These results indicate that FUL proteins regulate ethylene biosynthesis through enhancing the expression of *ACS2* and *ACO1* during tomato fruit ripening.

## Discussion

### *FUL1* and *FUL2* regulate tomato fruit ripening in a functionally redundant manner

*FUL1* was first identified in tomato as a floral identity gene (Pnueli *et al.*, 1991), but the expression pattern of *FUL1* suggests a possible role in fruit development and ripening (Busi *et al.*, 2003; Eriksson *et al.*, 2004). Moreover, it has previously been reported that *MADS-RIN* directly regulates the expression of the *FUL1* gene (Fujisawa *et al.*, 2012; Martel *et al.*, 2011). These results suggested that *FUL1* might be a candidate gene for regulating tomato fruit ripening, an idea that is supported by the observation that a *FUL1* homologue plays a central role in bilberry fruit ripening (Jaakola *et al.*, 2010). However, it was previously reported that suppressing a single tomato *FUL* gene resulted in no obvious phenotype (see Supplementary Fig. S1 at *JXB* online), but the double knock-out of *FUL1* and *FUL2* led to obvious phenotypes, such as yellow ripe fruits (Bemer *et al.*, 2012), and severely perturbed ethylene production. These results suggest that *FUL1* and *FUL2* contribute to tomato fruit ripening in a redundant manner. Our data also suggest that the functions of *FUL1* and *FUL2* have diversified since *FUL2*, but not *FUL1*, was observed to affect style abscission and cell expansion. Transgenic plants over-expressing either *FUL1* or *FUL2* were generated and, interestingly, *FUL2* over-expressing plants showed pleiotropic effects, including altered fruit shape (Fig. 2), stem structure (Fig. 3), and pericarp structure (Fig. 4), but *FUL1* over-expressing plants had no obvious phenotypic change.

In the *Arabidopsis ful* mutant, both cell expansion and differentiation were affected in the carpel valves which have broader and rounder cells and fewer cell layers than those of the wild type (Gu *et al.*, 1998). These results imply that the FRUITFULL protein has conserved functions in different plant species with regard to regulating cell expansion, differentiation, and fruit ripening. Our data suggest that *FUL1* has lost some of the functions that have been retained by *FUL2*. This functional loss may be related to differences in protein structure. Leseberg *et al.* (2008) analysed the protein–protein interactions of 16 tomato MADS-box proteins with the MADS-domain removed and showed that FUL2 could interact with RIN, TM3, JOINTLESS, MBP18, MBP24, MBP21, MADS1, TAG1, and TM5, but FUL1 could only interact with TM3, JOINTLESS, and RIN. The K domain of MADS-box transcription factors is responsible for the specificity of the DNA-binding dimer formation and it is proposed that structural differences in the K domains of FUL1 and FUL2 may be responsible for their functional divergence.

### *FUL2* regulates the abscission of styles from tomato fruit

Following pollination and fertilization, the style normally withers and abscises from the fruit. However, it was observed that this process was inhibited in fruit from the *FUL2*-OE transgenic lines. The whole style of *FUL2*-OE failed to abscise during fruit set, resulting in fruit with a pointed tip at the blossom end (Fig. 2). This suggests that *FUL2* regulates style abscission from tomato fruit at an early developmental stage. Inhibition of style abscission in *FUL2*-OE plants led to a fruit pointed tip phenotype. Seven phenotypically similar mutants (nipple-tip (*n*), *n-2*, *n-3, n-4*, *persistent style* (*pst*)*, beaked* (*bk)*, and *bk-2*) have previously been reported in tomato (Barten *et al.*, 1994). Of these, *n* has been assigned to tomato chromosome 5 (Mutschler *et al.*, 1987), and *pst* and *bk* mapped to chromosomes 7 and 2, respectively (Rick and Butler, 1956), but none of the corresponding genes had been cloned at this time. Accordingly, *FUL2* is not a candidate gene for the *n*, or *bk* genes as it is located on chromosome 3. These results also show that development of the tomato pistil is regulated by multiple genes but more experiments are needed to elucidate any potential relationship between *FUL2* and other ‘nipple tip’ genes.

How does *FUL2* regulate tomato cell development? Previous studies showed that expansin proteins may function not only in fruit softening and organ abscission, but may also regulate organ size and morphology. The expansin gene *AtEXP10* has been shown to encode a cell-wall-loosening protein that regulates organ size, morphology, and abscission and suppression of *AtEXP10* led to shorter petioles and leaf blades and a reduction in petiole cell size (Cho and Cosgrove, 2000). Indeed, a thinner pericarp with smaller cell sizes of *FUL2*-OE tomato fruits was observed compared with the wild type, as well as suppressed expression of the expansin gene *SlEXP1* (Fig. 4E). Thus, FUL2 might affect tomato style abscission and cell growth through regulating the expression of expansin genes, and it is proposed that the repressed expression of different expansin genes might also contribute to the thinner pericarp, stem, and small leaf phenotypes of the *FUL2*-OE transgenic lines.

### FUL proteins, TAGL1 and MADS-RIN may form higher order complexes to regulate tomato fruit ripening

MADS domains can bind to the CArG-box sites in the promoter region of many genes (Messenguy and Dubois, 2003), and in dimers of MADS-box proteins, both of the MADS domains take part in binding to a single DNA site (Pellegrini *et al.*, 1995; Santelli and Richmond, 2000). The heterodimer of the MADS-box proteins MC and JOINTLESS show stronger CArG-box DNA motif binding ability than MC and JOINTLESS alone, and the DNA-binding specificities of the heterodimer may be different from the homodimers of MC and JOINTLESS. This suggests that the heterodimerization of MADS-box proteins may be important for their physiological functions. TAGL1 and FULs can interact with the MADS-RIN protein, and all of them play important roles in tomato fruit ripening. It may be that both TAGL1 and FULs can form heterodimers with MADS-RIN, or form higher order complexes to regulate fruit ripening through an enhanced CArG-box DNA motif binding ability or altered DNA-binding specificities, and defects in any one of them perturbs tomato fruit ripening.

Similar to the *rin* mutant, *TAGL1*-RNAi and *FUL1*/*FUL2*-RNAi transgenic fruit showed a complete block in ethylene production and repressed expression of *ACS2* (Barry *et al.*, 2000; Vrebalov *et al.*, 2002). This suggests that *TAGL1*, the two *FUL* genes and *RIN* may regulate ethylene biosynthesis through a similar, or even an identical pathway. It has been confirmed that RIN, TAGL1 and FULs can bind to the *ACS2* promoter directly (Ito *et al.*, 2008; Itkin *et al.*, 2009; Shima *et al.*, 2013), and the heterodimerization of RIN with TAGL1 may regulate tomato fruit ripening by enhancing the ability of RIN and TAGL1 to bind to the *ACS2* promoter. If this is true, FULs may also form heterodimers with RIN, and this heterodimerization may enhance the ability of the RIN and FULs proteins to interact with the *ACS2* promoter directly and compensate for the functional defect caused by the failed heterodimerization of RIN and TAGL1. However, it is known that knocking down the expression of either *TAGL1* or the two *FUL* genes in tomato represses the ethylene burst during fruit ripening, which suggests that the interactions of RIN and TAGL1, or RIN and FULs, may not result in functional complementation. This raises the question, how do these three genes regulate autocatalytic ethylene production during tomato fruit ripening? One hypothesis is that RIN, TAGL1, and FULs can form heterodimers to regulate the expression of their own specific ripening related genes, and they can also form D-FUL-E higher order complexes to regulate the expression of *ACS2* during tomato fruit ripening. Indeed, yeast three-hybrid assays have shown that TAGL1, RIN, and FUL1 can form D-FUL-E higher order complexes (Leseberg *et al.*, 2008). Thus, the regulation of gene expression during fruit development and ripening by MADS box transcription factors is highly complex and much still remains to be learnt about the mode of action of *RIN* in regulating fruit ripening.

## Supplementary data

Supplementary data can be found at *JXB* online.

Supplementary Table S1. Primers used in this study.

Supplementary Fig. S1. *FUL1* and *FUL2* regulated fruit development and ripening.

Supplementary Data
