# Supplementary material for: Members of the tomato FRUITFULL MADS-box family regulate style abscission and fruit ripening
Source: J Exp Bot. 2014 Apr 10;65(12):3005–14. doi: 10.1093/jxb/eru137 (PMC4071821; doi:10.1093/jxb/eru137)
Supplement: Supplementary Data [file supp_65_12_3005__index.html]

Members of the tomato FRUITFULL MADS-box family regulate style abscission and fruit ripening — Members of the tomato FRUITFULL MADS-box family regulate style abscission and fruit ripening — Supplementary Data 

# Members of the tomato *FRUITFULL* MADS-box family regulate style abscission and fruit ripening

## Supplementary Data

Data files

**Files in this Data Supplement:**

- Supplementary Data - Supplementary Data
