# Supplementary material for: Members of the tomato FRUITFULL MADS-box family regulate style abscission and fruit ripening
Source: J Exp Bot. 2014 Apr 10;65(12):3005–14. doi: 10.1093/jxb/eru137 (PMC4071821; doi:10.1093/jxb/eru137)
Supplement: Supplementary Data [file supp_eru137_jexbot107813_file001.pdf]

Table S1 Genes encoding RIN-MIK interacting proteins identified from cDNA library of mixtures of immature green, mature green, breaker, and red ripe stages tomato fruit by yeast two-hybrid screening

Tomato gene ID	Gene name	Number of isolated clones	Annotation
Solyc06g069430	*FUL1*/*fruitfull1*/ *TDR4*/*TM4*	2	MADS box transcription factor
Solyc03g114830	*FUL2*/*fruitfull2*/ *SlMBP7*	5	MADS box transcription factor
Solyc09g010670	Unknown	2	DP-2 transcription factor-like
Solyc02g067030	*LeSNF1*	3	5'-AMP-activated protein kinase catalytic
Solyc10g081170	*calm2*	2	Calmodulin-2
Solyc04g045340	Unknown	2	Phosphoglucomutase
Solyc01g105340	Unknown	4	Chaperone protein dnaJ
Solyc06g076940	Unknown	1	NudC domain-containing
Solyc09g075950	Unknown	1	Heat shock protein 1
Solyc03g113020	Unknown	1	Vacuolar protein sorting 29
Solyc12g035130	Unknown	1	ATP dependent RNA helicase

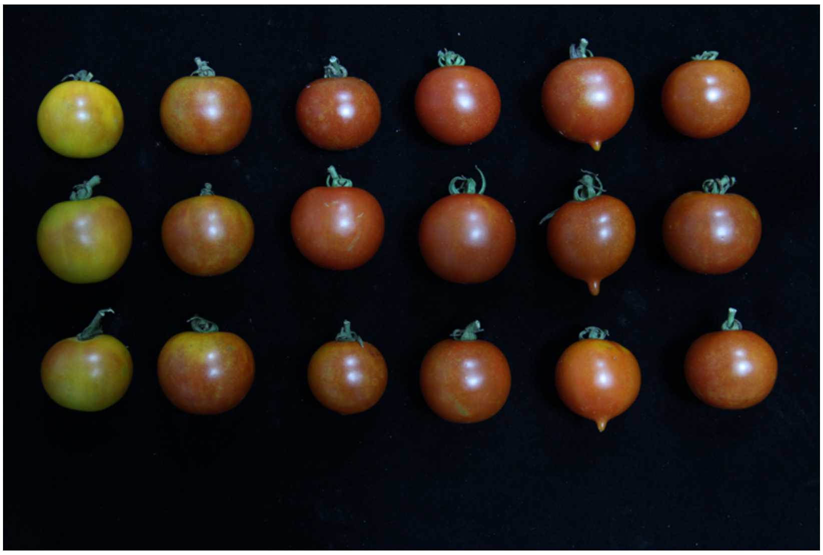

Figure S1. *FUL1* and *FUL2* regulated fruit development and ripening. Column 1, *FUL1/FUL2* RNAi fruits; column 2, *FUL1* RNAi fruits; column 3, *FUL2* RNAi fruits; column 4, wild-type fruits; column 5, *FUL2*-OE fruits; column 6, *FUL1*-OE fruits. All fruits were photographed at the BR+6 stage.
